# DNA typing from vaginal smear slides in suspected rape cases

**DOI:** 10.1590/S1516-31802004000200008

**Published:** 2004-03-01

**Authors:** Dayse Aparecida da Silva, Andréa Carla de Souza Góes, Jorge José de Carvalho, Elizeu Fagundes de Carvalho

**Keywords:** DNA Profile, Rape, Forensic sciences, Vaginal smears, Estupro, Esfregaço vaginal

## Abstract

In an investigation of suspected rape, proof of sexual assault with penetration is required. In view of this, detailed descriptions of the genitalia, the thighs and pubic region are made within the forensic medical service. In addition, vaginal swabs are taken from the rape victim and some of the biological material collected is then transferred to glass slides. In this report, we describe two rape cases solved using DNA typing from cells recovered from vaginal smear slides.

In 1999, two young women informed the Rio de Janeiro Police Department that they had been victims of sexual assaults. A suspect was arrested and the victims identified him as the offender. The suspect maintained that he was innocent. In order to elucidate these crimes, vaginal smear slides were sent to the DNA Diagnostic Laboratory for DNA analysis three months after the crimes, as unique forensic evidence. To get enough epithelial and sperm cells to perform DNA analysis, we used protocols modified from the previously standard protocols used for DNA extraction from biological material fixed on glass slides. The quantity of cells was sufficient to perform human DNA typing using nine short tandem repeat (STR) *loci*. It was 3.3 billion times more probable that it was the examined suspect who had left sperm cells in the victims, rather than any other individual in the population of Rio de Janeiro.

## RESUMO

Na suspeita de casos de estupro, é necessária a realização de exames para constatar a prática do abuso sexual com penetração. Com esse objetivo, nos serviços de medicina legal, é realizada, entre outros exames, a feitura de esfregaços vaginais com a transferência do material biológico para lâmina. Neste trabalho, são descritos dois casos forenses, elucidados pela tipagem de DNA realizada a partir de células de esfregaço vaginal.

Em 1999, duas jovens relataram ao Departamento de Polícia do Rio de Janeiro que haviam sido vítimas de abuso sexual. Um suspeito foi detido e apontado pelas vítimas como o agressor. Apesar das acusações, o suspeito alegou sua inocência. Com objetivo de elucidar os fatos, lâminas de esfregaço vaginal representando as únicas evidências biológicas dos suspeitosos casos de estupro foram enviadas ao Laboratório de Diagnósticos por DNA três meses após o crime. Os protocolos utilizados para recuperação celular e extração de DNA baseiam-se em técnicas preexistentes, porém adaptadas para se obter quantidade de DNA suficiente para realização das análises. A quantidade de células foi suficiente para a utilização da técnica de extração diferencial de DNA e sua tipagem por meio de 9 *loci* "short tandem repeats" (STR). A análise estatística mostrou ser 3,3 bilhões de vezes mais provável que o relatado suspeito seja o doador das células espermáticas encontradas no material biológico coletado das vítimas que qualquer outro indivíduo na população do Rio de Janeiro.

## INTRODUCTION

In the State of Rio de Janeiro, more than 1,000 rape cases are brought to the attention of official investigators per year.^1^ Rape is the crime of forced sexual intercourse, against the victim's will. There are many reasons why rape victims may not able to identify the perpetrator. On the other hand, in a small number of cases, the victim can identify the offender, but the suspect can easily refute the accusation. In the light of this, analysis of the evidence from crimes of sexual assault can lead to vital information in the process of identifying the assailant.

Techniques for individual identification have now been developed on the basis of DNA analysis, with high discrimination potential and sensitivity.^2^ DNA typing should therefore be conducted to identify the offender(s) from among the indicated suspects.

Before applying the human DNA typing methodology, swabs taken from the victim are smeared onto glass slides for sperm cell screening via optical microscopy. The presentation of vaginal smears on glass slides is not a routine procedure for DNA typing purposes in rape cases. Thus, additional swabs should be collected from the victim as the possible source of the suspect's biological material.^3^

In spite of this recommendation, we have faced the challenge of obtaining DNA in high quality and enough quantity from cells on vaginal smear slides since 1999, when DNA analysis was employed for the first time in the State of Rio de Janeiro to solve criminal cases.^4^ Multiplex autosomal short tandem repeats (STRs) at present represent the most popular approach to forensic DNA analysis. Short tandem repeat *loci* consist of three to seven base pair sequences, repeated in tandem.^5^ They provide a rich source of polymorphic markers resulting from variation in the number of copies of the repeat motif. STRs are usually genotyped via the polymerase chain reaction (PCR), a technique that amplifies DNA by means of a series of thermal cycles. Genotypes may be detected simply by silver staining after gel electrophoresis of the PCR product or via fluorescence technologies.^6^

In this report, we describe two forensic cases solved at the DNA Diagnostic Laboratory of Universidade Estadual do Rio de Janeiro, using DNA extracted from vaginal smear slides. In order to obtain sufficient quantity of epithelial and sperm cells, we used a methodology that represents an improvement on the standard protocols used for DNA extraction from biological material fixed on glass slides.^7-9^

## METHODS

Initially, the smear slides were immersed in xylene at 56° C, for 30 minutes, and then in absolute ethanol for five minutes, 70% etha- nol/1 mM Tris HCl pH 7.4 for five minutes, and sterile phosphate buffer saline (2.7 mM KCl; 137 mM NaCl; 1.5 mM KH_2_PO_4_/Na_2_HPO_4_; pH 7.4) for ten minutes. Following this, biological material representing half of the vaginal smear slide was transferred to a 1.5-ml microcentrifuge tube and centrifuged at 12,000 G, at room temperature, for five minutes. The cell pellet was resuspended in 20 µl of phosphate buffer saline.

The isolation of sperm DNA from the mixture of spermatozoa and vaginal epithelial cells was based on differential lysis.^10^ The DNA of the male and female fractions was purified using phenol-chloroform-isoamyl alcohol (25:24:1) (Life Technologies) and concentrated by ultra filtration using 100,000 molecular weight cutoff filters (Microcon 100, Amicon). The DNA pellet was resuspended in 10 µl TE (1 mM TrisHCl, 0.5 mM EDTA). DNA was extracted from blood and saliva samples by organic extraction methods and ethanol precipitation according to Comey et al.^10^ DNA quantification was carried out by measuring optical densities at 260 nm.

2 ng of template DNA were added to a 25-µl amplification reaction using primers that were set to nine short tandem repeat *loci* (Table 1), which were included in the multiplex silver-staining systems from Promega Corporation.^11^

**Table 1:** Features of individual short tandem repeat (STR) *loci* and forensic case genotype information

Loci *Information*			*Forensic case information*							
				Case 1			Case 2			
	*(%)*	*Heterozygosity*	S	Ev1		V1	Ev2		V2	f
*Locus* (gene bank name)	Chromosome location	Rio de Janeiro Population		MF	FF		MF	FF		
CSF1PO (HUMCSF1PO)	5q33.5-34	70.19	10 12	10 12	8 10	8 10	10 12	8 8	8 8	0.189
TPOX (HUMTPOX)	2p23-2p	68.27	8 9	8 9	8 12	8 12	8 9	11 13	11 13	0.093
THO1 (HUMTHO1)	11p15.5	75.96	6 7	6 7	7 9,3	7 9,3	6 7	5 5	5 5	0.090
F13A01 (HUMF13A01)	6p24-25	77.52	4 5	4 5	4 12	4 12	4 5	10 11	10 11	0.034
FESFPS (HUMFESFPS)	15q25-qter	80.00	9 11	9 11	10 11	10 11	911 11	6 9.3	6 9.3	0.062
VWA (HUMVWFA31)	12p12-pter	76.92	17 18	17 18	18 19	18 19	17 18	15 16	15 16	0.077
D16S539	16q24-qter	81.73	11 12	11 12	10 11	10 11	11 12	12 13	12 13	0.148
D7S820	7q11.21-q22	88.46	10 12	10 12	8 11	8 11	10 12	10 13	10 13	0.093
D13S317	13q22-q31	75.96	8 11	8 11	8 12	8 12	8 11	11 12	11 12	0.087
					CCI = 1 in 3,300,000,000					

***S:*** *suspect's DNA profile for nine STR* loci; ***Ev1 (MF/FF):*** *male and female fraction profiles from vaginal smear slide cells (Ev1: biological evidence from Case 1); **V1:** victim's DNA profile for nine STR* loci *(Case 1); **Ev2 (MF/FF):** Male and female profiles from vaginal smear slide cells (Ev2: biological evidence from Case 2); **V2:** victim's DNA profile for nine STR* loci *(Case 2); **f:** suspect's genotype frequency; **CCI:** cumulative concordance index (matching probability): the average number of individuals that would have to be surveyed to find a match among randomly selected individuals. The CCI calculation was done using a Rio de Janeiro population genotype data bank (Universidade Estadual do Rio de Janeiro/DNA Diagnostic Laboratory).*

Electrophoretic analysis of the polymerase chain reaction products was performed using a 4% polyacrylamide denaturing gel. Gels underwent electrophoresis for 1.5 hours at constant power (30 W). Following electrophoresis, the analytical gel was submitted to silver staining.^6^ A data bank for the population of the State of Rio de Janeiro was utilized.^12^

## RESULTS

**Figure 1:**
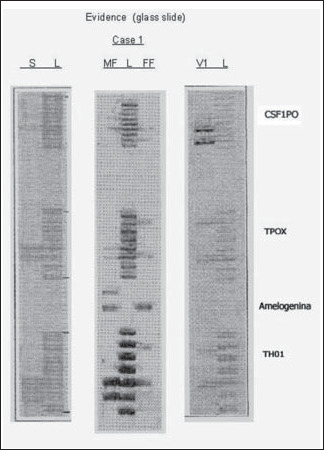
DNA profiling in Case 1. The amplification products for the CTT (CSF1PO, TPOX and TH01) multiplex in polymerase chain reactions carried out using DNA from the suspect and victim. Male and female fractions from vaginal smear slides underwent electrophoresis in a 4% polyacrylamide denaturing gel. Alleles were detected by silver stain analysis. Lane L: STR DNA marker ladder; lane S: suspect's saliva DNA; lane MF: vaginal swab (male fraction); lane FF: vaginal swab (female fraction); lane V1: victim's blood DNA.

The results for the CSF1PO, TPOX and TH01 *loci* are shown in Figure 1. It can be seen that the DNA profiles obtained for the male and female fractions from the vaginal smear slides correspond to those from the suspect and victim, respectively. In addition to the CSF1PO, TPOX and TH01 typed alleles, DNA profiling for the six short tandem repeat *loci* (D16S539, D7S820, D13S317, F13A01, FESFPS and vWA) and the statistics for each case are summarized in Table 1.

The DNA profiles observed for the spermatic DNA fractions from the vaginal smear slides from both victims were identical, thereby indicating that the rapes were committed by a single person. The spermatozoa DNA profile also matched that of the suspect. With regard to the statistics, on the basis of DNA analysis for nine STR *loci*, it was 3.3 billion times more probable that it was the examined suspect who had left sperm cells in the victims, rather than any other individual in the population.

## DISCUSSION

In the investigation of cases of sexual assault, the collection of additional swabs for performing DNA analysis is strongly recommended. Unfortunately, in Brazilian forensic medical services, vaginal or oral swabs are not routinely taken for DNA analysis. Thus, only the biological evidence fixed on glass slides is available for investigations using DNA typing technology for identifying suspects' DNA profiles.

The biological material collected from victims' anal and vaginal cavities is usually fixed on slides by alcohol, stained using the Shorr technique and mounted with cover slide using balsamic resins (Canada balsam). For DNA analysis, the cover slip and balsamic resin must first of all be taken away, in order to gain access to the biological material. This can be achieved using xylene. In addition, it is strongly recommended that all traces of xylene and dyes should then be removed. It has been reported that dyes coextracted with the DNA can reduce the efficiency of the polymerase chain reaction.^13^

With regard to these two rape cases, it has been shown that the DNA obtained from the vaginal smear slides, using the methodology described in this work, was adequate for performing human DNA typing with confidence, thereby allowing reliable and interpretable results to be applied during routine forensic analysis.
